# Supplementary material for: Modeling glioblastoma heterogeneity as a dynamic network of cell states
Source: Mol Syst Biol. 2021 Sep 16;17(9):e10105. doi: 10.15252/msb.202010105 (PMC8444284; doi:10.15252/msb.202010105)
Supplement: Supplementary file 6 — Source Data for Figure 5 [file MSB-17-e10105-s004.zip › Figure5A_sourcedata/GSEA_3071/hallmarks_stateV.GseaPreranked.1623742546329/HALLMARK_HYPOXIA.html]

Details for gene set HALLMARK\_HYPOXIA[GSEA]

|  || Dataset | state43071 |
| Phenotype | NoPhenotypeAvailable |
| Upregulated in class | na\_neg |
| GeneSet | HALLMARK\_HYPOXIA |
| Enrichment Score (ES) | -0.48134813 |
| Normalized Enrichment Score (NES) | -2.6776822 |
| Nominal p-value | 0.0 |
| FDR q-value | 0.0 |
| FWER p-Value | 0.0 |
Table: GSEA Results Summary

  

Fig 1: Enrichment plot: HALLMARK\_HYPOXIA      
 Profile of the Running ES Score & Positions of GeneSet Members on the Rank Ordered List

  

| PROBE | GENE SYMBOL | GENE\_TITLE | RANK IN GENE LIST | RANK METRIC SCORE | RUNNING ES | CORE ENRICHMENT || 1 | IGFBP3 |  |  | 78 | 1.437 | 0.0032 | No |
| 2 | CSRP2 |  |  | 90 | 1.396 | 0.0187 | No |
| 3 | PDGFB |  |  | 283 | 1.080 | -0.0046 | No |
| 4 | MAP3K1 |  |  | 288 | 1.077 | 0.0083 | No |
| 5 | HEXA |  |  | 341 | 1.024 | 0.0112 | No |
| 6 | CDKN1B |  |  | 360 | 0.997 | 0.0203 | No |
| 7 | CDKN1A |  |  | 363 | 0.993 | 0.0325 | No |
| 8 | NEDD4L |  |  | 413 | 0.939 | 0.0349 | No |
| 9 | SAP30 |  |  | 424 | 0.930 | 0.0448 | No |
| 10 | STC1 |  |  | 433 | 0.920 | 0.0549 | No |
| 11 | AKAP12 |  |  | 487 | 0.878 | 0.0558 | No |
| 12 | NCAN |  |  | 540 | 0.842 | 0.0564 | No |
| 13 | CXCR4 |  |  | 550 | 0.830 | 0.0652 | No |
| 14 | CHST3 |  |  | 579 | 0.813 | 0.0701 | No |
| 15 | GPC4 |  |  | 652 | 0.772 | 0.0660 | No |
| 16 | ENO2 |  |  | 728 | 0.731 | 0.0608 | No |
| 17 | PAM |  |  | 789 | 0.705 | 0.0582 | No |
| 18 | VEGFA |  |  | 926 | 0.649 | 0.0402 | No |
| 19 | VHL |  |  | 1047 | 0.609 | 0.0248 | No |
| 20 | SLC37A4 |  |  | 1195 | 0.564 | 0.0037 | No |
| 21 | IRS2 |  |  | 1400 | 0.505 | -0.0292 | No |
| 22 | HK1 |  |  | 1611 | 0.458 | -0.0638 | No |
| 23 | DPYSL4 |  |  | 1657 | 0.444 | -0.0668 | No |
| 24 | CCNG2 |  |  | 1780 | 0.422 | -0.0849 | No |
| 25 | WSB1 |  |  | 1784 | 0.421 | -0.0802 | No |
| 26 | SCARB1 |  |  | 1920 | 0.401 | -0.1011 | No |
| 27 | LARGE1 |  |  | 2006 | 0.387 | -0.1125 | No |
| 28 | VLDLR |  |  | 2236 | 0.358 | -0.1520 | No |
| 29 | PFKL |  |  | 2241 | 0.358 | -0.1483 | No |
| 30 | ZNF292 |  |  | 2267 | 0.354 | -0.1486 | No |
| 31 | TPD52 |  |  | 2318 | 0.347 | -0.1539 | No |
| 32 | FOS |  |  | 2529 | 0.319 | -0.1902 | No |
| 33 | SRPX |  |  | 2646 | 0.305 | -0.2087 | No |
| 34 | BCL2 |  |  | 2680 | 0.302 | -0.2112 | No |
| 35 | PDK3 |  |  | 2707 | 0.298 | -0.2124 | No |
| 36 | TIPARP |  |  | 2768 | 0.292 | -0.2203 | No |
| 37 | CHST2 |  |  | 3126 | 0.258 | -0.2857 | No |
| 38 | SIAH2 |  |  | 3142 | 0.256 | -0.2853 | No |
| 39 | RORA |  |  | 3171 | 0.253 | -0.2875 | No |
| 40 | PGK1 |  |  | 3286 | -0.263 | -0.3061 | No |
| 41 | IDS |  |  | 3288 | -0.264 | -0.3030 | No |
| 42 | PRDX5 |  |  | 3296 | -0.266 | -0.3010 | No |
| 43 | BNIP3L |  |  | 3381 | -0.278 | -0.3136 | No |
| 44 | HMOX1 |  |  | 3426 | -0.286 | -0.3185 | No |
| 45 | GCNT2 |  |  | 3439 | -0.288 | -0.3171 | No |
| 46 | ERO1A |  |  | 3506 | -0.300 | -0.3260 | No |
| 47 | BHLHE40 |  |  | 3620 | -0.318 | -0.3437 | No |
| 48 | TNFAIP3 |  |  | 3691 | -0.330 | -0.3530 | No |
| 49 | NFIL3 |  |  | 3700 | -0.331 | -0.3504 | No |
| 50 | MAFF |  |  | 3849 | -0.359 | -0.3743 | No |
| 51 | JUN |  |  | 4135 | -0.418 | -0.4238 | No |
| 52 | LDHA |  |  | 4145 | -0.421 | -0.4203 | No |
| 53 | DDIT4 |  |  | 4182 | -0.432 | -0.4217 | No |
| 54 | STC2 |  |  | 4358 | -0.488 | -0.4492 | No |
| 55 | HDLBP |  |  | 4464 | -0.524 | -0.4628 | No |
| 56 | HOXB9 |  |  | 4498 | -0.540 | -0.4623 | No |
| 57 | UGP2 |  |  | 4526 | -0.553 | -0.4605 | No |
| 58 | ADORA2B |  |  | 4535 | -0.556 | -0.4550 | No |
| 59 | NR3C1 |  |  | 4673 | -0.630 | -0.4734 | Yes |
| 60 | P4HA1 |  |  | 4689 | -0.641 | -0.4682 | Yes |
| 61 | HSPA5 |  |  | 4714 | -0.652 | -0.4645 | Yes |
| 62 | PFKP |  |  | 4734 | -0.666 | -0.4598 | Yes |
| 63 | SLC2A1 |  |  | 4739 | -0.671 | -0.4521 | Yes |
| 64 | PIM1 |  |  | 4783 | -0.699 | -0.4515 | Yes |
| 65 | MT2A |  |  | 4799 | -0.714 | -0.4454 | Yes |
| 66 | PNRC1 |  |  | 4813 | -0.726 | -0.4387 | Yes |
| 67 | MIF |  |  | 4900 | -0.811 | -0.4450 | Yes |
| 68 | TGM2 |  |  | 4928 | -0.842 | -0.4395 | Yes |
| 69 | KLF6 |  |  | 4933 | -0.849 | -0.4295 | Yes |
| 70 | SLC2A3 |  |  | 4942 | -0.859 | -0.4202 | Yes |
| 71 | PPP1R15A |  |  | 4966 | -0.882 | -0.4135 | Yes |
| 72 | KDELR3 |  |  | 4974 | -0.893 | -0.4036 | Yes |
| 73 | TMEM45A |  |  | 4980 | -0.897 | -0.3932 | Yes |
| 74 | ENO1 |  |  | 4999 | -0.930 | -0.3849 | Yes |
| 75 | CITED2 |  |  | 5045 | -1.026 | -0.3806 | Yes |
| 76 | ANXA2 |  |  | 5055 | -1.036 | -0.3692 | Yes |
| 77 | ACKR3 |  |  | 5065 | -1.065 | -0.3575 | Yes |
| 78 | MYH9 |  |  | 5090 | -1.125 | -0.3479 | Yes |
| 79 | LOX |  |  | 5092 | -1.128 | -0.3338 | Yes |
| 80 | CAVIN1 |  |  | 5116 | -1.195 | -0.3231 | Yes |
| 81 | GLRX |  |  | 5121 | -1.212 | -0.3086 | Yes |
| 82 | TGFBI |  |  | 5136 | -1.259 | -0.2953 | Yes |
| 83 | DUSP1 |  |  | 5138 | -1.269 | -0.2795 | Yes |
| 84 | MT1E |  |  | 5154 | -1.323 | -0.2656 | Yes |
| 85 | SERPINE1 |  |  | 5161 | -1.377 | -0.2494 | Yes |
| 86 | P4HA2 |  |  | 5164 | -1.393 | -0.2322 | Yes |
| 87 | DDIT3 |  |  | 5180 | -1.461 | -0.2166 | Yes |
| 88 | TPST2 |  |  | 5182 | -1.472 | -0.1982 | Yes |
| 89 | PLAUR |  |  | 5185 | -1.479 | -0.1798 | Yes |
| 90 | BGN |  |  | 5216 | -1.654 | -0.1647 | Yes |
| 91 | TPBG |  |  | 5229 | -1.755 | -0.1448 | Yes |
| 92 | EGFR |  |  | 5230 | -1.765 | -0.1225 | Yes |
| 93 | DCN |  |  | 5233 | -1.798 | -0.1001 | Yes |
| 94 | S100A4 |  |  | 5238 | -1.817 | -0.0779 | Yes |
| 95 | F3 |  |  | 5242 | -1.927 | -0.0542 | Yes |
| 96 | COL5A1 |  |  | 5274 | -2.455 | -0.0291 | Yes |
| 97 | CAV1 |  |  | 5279 | -2.605 | 0.0031 | Yes |
Table: GSEA details [plain text format]

  

Fig 2: HALLMARK\_HYPOXIA: Random ES distribution      
 Gene set null distribution of ES for **HALLMARK\_HYPOXIA**
